# Visualizing 50 Years of Cancer Mortality Rates Across the US at Multiple Geographic Levels Using a Synchronized Map and Graph Animation

**DOI:** 10.5888/pcd17.190286

**Published:** 2020-03-26

**Authors:** Isaac H. Michaels, Sylvia J. Pirani, Alvaro Carrascal

**Affiliations:** 1 Department of Epidemiology and Biostatistics, University at Albany School of Public Health, Rensselaer, New York; 2 Health Resources and Services Administration Region 2 Public Health Training Center, Columbia University Mailman School of Public Health, Department of Sociomedical Sciences, New York, New York

**Figure:**
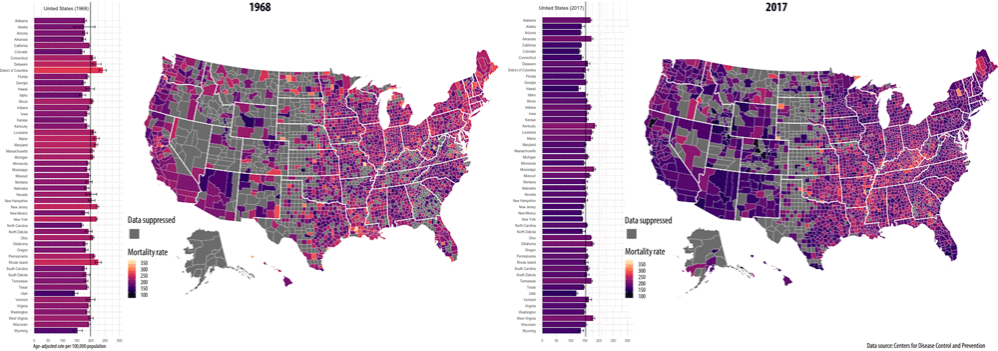
Static display of the change in US cancer mortality rates from 1968 to 2017.

We developed the synchronized map and graph animation at https://www.youtube.com/embed/5sGU5hH-0eA to visualize changes over time in yearly, age-adjusted, cancer mortality rates at the county, state, and national geographic levels for the United States from1968 through 2017. The goal was to enable viewers to select trends of interest for a particular state, region, or time interval.

## Background

Cancer is the second leading cause of death in the United States (1). An estimated 42% of all cancer cases and nearly one-half of all cancer deaths in the United States are attributable to modifiable risk factors (2). Health officials and stakeholders need visualizations of data on cancer deaths to target prevention and treatment efforts optimally.

One way of showing changes over time in spatial data is to present side-by-side maps, each map representing data for a different time during the period (3). Animations have been used to present changes over time more granularly than static maps (4). To improve this method and accommodate data for multiple geographic levels, our project proposed a novel technique for visualizing temporal trends in spatial data — presenting an animated choropleth (thematic) map alongside a synchronized animated horizontal bar chart. We demonstrated the method by using data on cancer deaths from 1968 through 2017 in the United States at the county, state, and national geographic levels.

## Data Sources and Map Logistics

Age-adjusted mortality rates, stratified by year, for counties, individual states, and the United States were obtained from the CDC WONDER (Wide-ranging Online Data for Epidemiologic Research) website, including its Compressed Mortality File 1968 through 1978, its Compressed Mortality File 1979 through 1998, and its Multiple Cause of Death file for 1999 through 2017 (5). Cancer deaths were defined as deaths with any malignant cancer listed as the underlying cause. Malignant cancer was indicated by the *International Classification of Diseases* (ICD-8) codes 140–207 during 1968 through 1978, by ICD-9 codes 140–208 and 238.6 during 1979 through 1998, and by ICD-10 codes C00–C97 during 1999 through 2017 (6). Three ICD case definitions were necessary to compare data for all years during 1968 through 2017 (Table). The possibility of sensitivity or specificity differing among the case definitions is, therefore, a limitation of this project.

**Table:** Cancer Deaths, United States, 1968–2017^a^

Year	Deaths	Age-Adjusted Rate per 100,000 Population
2017	599,108	152.5
2016	598,038	155.8
2015	595,930	158.5
2014	591,700	161.2
2013	584,881	163.2
2012	582,623	166.5
2011	576,691	169.0
2010	574,743	172.8
2009	567,628	173.5
2008	565,469	176.4
2007	562,875	179.3
2006	559,888	181.8
2005	559,312	185.1
2004	553,888	186.8
2003	556,902	190.9
2002	557,271	194.3
2001	553,768	196.5
2000	553,091	199.6
1999	549,838	200.8
1998	541,582	200.8
1997	539,615	203.5
1996	539,593	206.8
1995	538,505	209.9
1994	534,353	211.8
1993	529,951	213.5
1992	520,616	213.5
1991	514,705	215.2
1990	505,366	216.0
1989	496,202	214.2
1988	485,082	212.5
1987	476,965	211.8
1986	469,411	211.6
1985	461,606	211.3
1984	453,530	210.8
1983	443,020	209.1
1982	433,833	208.4
1981	422,132	206.4
1980	416,525	207.9
1979	403,427	204.0
1978	395,149	203.9
1977	384,905	202.5
1976	375,687	201.6
1975	364,111	199.2
1974	358,961	200.6
1973	349,597	199.2
1972	344,130	199.4
1971	336,005	198.5
1970	329,433	197.8
1969	321,804	197.7
1968	317,465	198.1

a Data sources: Centers for Disease Control and Prevention, National Center for Health Statistics, Compressed Mortality File 1968–1978 and Compressed Mortality File 1979–1998 and CDC WONDER Online Database, Multiple Cause of Death Files 1999–2017 (5). International Classification of Diseases (ICD) definitions: ICD-8 codes 140–207 during 1968–1978; ICD-9 codes 140–208 and 238.6 during 1979–1998; and ICD-10 codes C00–C97 during 1999–2017 (6).

We developed and animated a horizontal bar graph and a choropleth map in R version 3.6.0 (R Foundation for Statistical Computing) (7), by using the albersusa (8), ggplot2 (9), viridis (10), ggthemes (11), gganimate (12), and magick (13) packages. Graph and map animations were rendered separately as graphics interchange format (GIF) images, and then combined. The open-source FFmpeg software suite was used to convert the animated GIF image into an MP4 formatted video (14).

## Highlights

The animation has a short duration, which facilitates consecutive viewings. The animation also leverages interactive features of MP4 video players, such as play, pause, vary playback speed, advance frame-by-frame, rewind, fast forward, and jump to specific places. These constitute a partial menu of possible interactive features that a data visualization might incorporate. We acknowledge that, in this sense, our animated data visualization has limitations. The combined animation’s interactivity, open layout, and high placement of the title; however, are consistent with common design practices for animated maps online that generally conform to cartographic standards (15).

## Action

Our animated data visualization presents cancer mortality rates spatially and temporally and illustrates that despite the overall decrease nationally in the age-adjusted rate from 1968 through 2017, disparities persisted among states and counties. This visualization can be used to improve public health resource targeting and evidence-based intervention efforts for states and counties with emerging or persistently high cancer mortality rates. Health officials, policy makers, and stakeholders can use data animation to inform policies and practices that influence cancer outcomes. For example, animation can focus attention on counties throughout the Mississippi Delta and Appalachia, where declines in cancer mortality have lagged compared with national declines — a known pattern that would be difficult to discern from a static data visualization.

Animated choropleth mapping is a novel visualization method for health data. Our project is the first, of which we are aware, to combine an animated graph and animated choropleth map. Although specific data, such as maximum and minimum values, might be difficult to convey by animated visualizations, animation can be effective for adding another dimension of information, particularly time, to static data visualizations. In doing so, animation can enable some data visualizations to convey patterns and relationships that are not apparent from static visualizations, especially across geographic levels. Therefore, we encourage data analysts to consider synchronized graph and choropleth map animations as an option for communicating data to public health researchers, practitioners, and policy makers.
